# Psychological Health Issues Subsequent to SARS-Cov 2 Restrictive Measures: The Role of Parental Bonding and Attachment Style

**DOI:** 10.3389/fpsyt.2020.589444

**Published:** 2020-11-04

**Authors:** Silvia Bussone, Chiara Pesca, Renata Tambelli, Valeria Carola

**Affiliations:** ^1^Department of Dynamic and Clinical Psychology, Sapienza University of Rome, Rome, Italy; ^2^Experimental Neuroscience, Santa Lucia Foundation IRCCS-Rome, Rome, Italy

**Keywords:** SARS-CoV 2, COVID-19, clinical psychology, SCL-90-R, perceived stress, state anxiety, attachment, parental bonding (PBI)

## Abstract

**Background:** The novel coronavirus 2019 (COVID-19) has caused severe panic among people worldwide. In Italy, a nationwide state of alert was declared on January 31st, leading to the confinement of the entire population from March 11 to May 18, 2020. Isolation and quarantine measures cause psychological problems, especially for individuals who are recognized as being vulnerable. Parental bonding and attachment styles play a role in the programming of the stress response system. Here, we hypothesize that the response to restricted social contact and mobility due to the pandemic has detrimental effects on mental-psychological health and that this relationship is, at least in part, modulated by parental bonding and attachment relationships that are experienced at an early age.

**Methods:** A sample of 68 volunteer University students was screened for psychopathological symptoms (SCL-90-R and STAI-Y), stress perception (PSS), attachment style (RQ), and parental care and overcontrol (PBI) 6 months before the confinement. In the same subjects, psychopathological symptoms and stress perception were measured again during confinement.

**Results:** Overall, psychological health and stress management deteriorated across the entire sample during confinement. Specifically, a significant increase in phobic anxiety, depression, psychological distress, and perceived stress was observed. Notably, parental bonding and attachment styles modulated the psychological status during the lockdown. Individuals with secure attachment and high levels of parental care (high care) showed increased levels of state anxiety and perceived stress in phase 2, compared with phase 1. In contrast, individuals with insecure attachment and low levels of parental care (low care) already showed a high rate of state anxiety and perceived stress in phase 1 that did not increase further during phase 2.

**Conclusion:** The general deterioration of psychological health in the entire sample demonstrates the pervasiveness of this stressor, a decline that is partially modulated by attachment style and parental bonding. These results implicated disparate sensitivities to environmental changes in the high- and low care groups during the lockdown, the former of which shows the greatest flexibility in the response to environment, suggesting adequate and functional response to stress in high care individuals, which is not observable in the low care group.

## Introduction

Responsivity and adaptability to stress are among the main risk factors for psychopathologies. Fundamentally, these mechanisms favor an individual's adaptation to a changing environment, promoting survival (1–3). The stress responsivity system is designed to coordinate responses to psychosocial stressors, filter environmental information to coordinate behavioral responses, and regulate behavioral responses, based on an individual's life history and behavioral repertoire (2). An event can be categorized as stressful when an individual perceives the environmental demands to exceed his adaptive capacity (4). In psychology, perceived stress is a concept connected with the individual's feelings about the general stressfulness of his life and his ability to handle such stress rather than a feeling measuring the frequencies of stressful events that happen to a person. It is generally believed that perceived stress influences both physical and psychological health status (5). Notably, the frequency of serious psychological symptoms, such as depression and anxiety, has been shown to be related either directly (6–8) or indirectly (9, 10) to perceived stress.

Stress reactivity is programmed by early-life experiences (3), such as those that engage motivational systems, such as parental care and attachment (11). Notably, low levels of parental care, reflecting cold, distant parent-child relationships, are associated with a significantly increased risk for depression and anxiety in adulthood [e.g., (12–15)]. Exposure to such adverse environmental conditions at an early age alters the development of the ability to cope with the stress (16). Similar findings have been reported in preclinical studies, in which the centrality of early-life experiences, such as maternal care, in the maturation and development of the behavioral and physiological responsivity to stress, has been postulated (16, 17).

Exposure to epidemics is a stressful event that impacts the entire population. In addition to affecting an individual's physical health, it has many implications for mental health. On an individual level, people experience fear of becoming sick or dying from an epidemic or infectious disease, feelings of helplessness, and stigma under these conditions (18). Moreover, social isolation that is associated with quarantine frequently catalyzes many mental health sequelae (19). However, epidemic-induced stress and psychopathological symptoms are likely to vary among individuals in relation to the individual's ability to cope with stress, an ability derived from early-life experiences such as parental care and attachment. It has been indeed reported that the susceptibility to the development of neuroticism in presence of SARS epidemics was modulated by parental attachment, particularly attachment to the mother (20).

The ongoing COVID-19 pandemic is inducing fear worldwide, and preliminary evaluations and reports on its consequences describe its impact as transversal, affecting anyone (21). Specifically, 25% of the general population has experienced moderate to severe stress- or anxiety-related symptoms in response to COVID-19 (22, 23). Thus, a timely understanding of its impact on mental health is urgently needed (24). Moreover, a recent study describing the modulatory role of attachment styles on psychological distress due to COVID-19 exposure (25), supports the necessity of clarifying the role of attachment and parental care in modulating the physiological and psychological response to this epidemic.

In Italy, a nationwide state of alert was declared on January 31, 2020, leading to the confinement of the entire population from March 11 to May 18, 2020. As a consequence of this pandemic, strict measures of social isolation and social distancing have been implemented.

We hypothesized that the response to the social isolation and restrictive measures due to the COVID-19 pandemic has had detrimental effects on mental health and that the response to this event is, at least in part, modulated by the stressful life events that an individual experienced at an early age, contributing to the development of his ability to cope with stress.

## Materials and Methods

### Participants

Participants included a group of 68 volunteer University students (10 men and 58 women; mean age ± SE = 2,490 ± 2,797 years). Prior to enrolment, all participants were given a complete description of the study and signed a written informed consent. The sample was divided into sub-groups according to attachment style measured by Relationship Questionnaire (secure, *N* = 24; insecure *N* = 44) and perceived parental care (low, *N* = 21; high, *N* = 24; intermediate, *N*= 23) and parental control (low, *N* = 34; high, *N* = 13; intermediate, *N* = 21) measured by Parental Bonding Instrument as described in the section below.

The study was approved by the Ethical Committee of the Department of Dynamic and Clinical Psychology, Sapienza, University of Rome (Prot. n. 0000453 and Prot. n. 0000112). None of the subjects received a COVID-19 diagnosis. When asked “who did you spend the quarantine with?” overall, the 13.2% of the sample spent the quarantine with their flat mates, the 16.2% with their partner, the 2% alone, and the remaining 46% with their family.

### Clinical Assessment

The online administration of the questionnaires was repeated at two time points (phase): (1) 6 months (on average) before the COVID-19 pandemic; and (2) during the last weeks of confinement/lockdown in Italy (from April 23 to May 4, 2020). Anamnestic information (about the individual's life conditions), psychopathological symptoms, perceived stress, attachment style, parental care, and parental control measurements were collected on the same subjects, at these time points. At phase 1, the screened sub-groups (secure vs. insecure; high vs. intermediate, and vs. low parental care/control) differed in symptom severity, perceived stress, and state anxiety (Supplementary Tables 1–4) measured, respectively by Symptom Check-List-90 item Revised questionnaire, Perceived Stress Scale-10, and State-Trait Anxiety Inventory, as described in the section below.

#### Symptom Check-List-90 Item Revised (SCL-90-R)

SCL-90-R is a 90-item self-report questionnaire, evaluating psychopathological symptoms and psychological distress in adults from general and clinical populations (26). The SCL-90-R is rated on a Likert scale of 0 (not at all) to 4 (extremely), and asks participants to report if they have suffered in the past week from symptoms of somatization (e.g., headaches), obsessive-compulsivity (e.g., having to check and double-check what you do), interpersonal sensitivity (e.g., feeling that people are unfriendly or dislike you), depression (e.g., feeling blue), anxiety (e.g., feeling fearful), hostility (e.g., having urges to beat, injure, or harm someone), phobic anxiety (e.g., feeling afraid to go out of your house alone), paranoid ideation (e.g., the idea that you should be punished for your sins), and psychoticism (e.g., having thoughts that are not your own). Aside from these nine primary scales, the questionnaire provides a global severity index (GSI), which is used to determine the severity and degree of psychological distress. The SCL-90-R showed good internal coherence (α = 0.88) in this study [Italian validated version (27)].

#### Perceived Stress Scale-10 (PSS)

PSS-10 (5) measures the degree to which one perceives aspects of one's life as uncontrollable, unpredictable, and over-loading. Participants are asked to respond to each question on a 5-point Likert scale ranging from 0 (never) to 4 (very often), indicating how often they have felt or thought a certain way within the past month. Scores range from 0 to 40, with higher composite scores indicative of greater perceived stress. The PSS-10 possesses adequate internal reliability (5) [Italian validated version (6)].

#### State-Trait Anxiety Inventory (STAI-Y)

STAI-Y (28) consists of 40 statements about the feelings of the participant, divided into two parts. In Part I (20 statements), volunteers are instructed to indicate the intensity of their feelings of anxiety at a moment (state anxiety), using scores ranging from 1 (absolutely not) to 4 (very much). In Part II (other 20 statements), volunteers describe how they generally feel (trait anxiety) by reporting the frequency of their symptoms of anxiety, again using scores ranging from 1 (hardly ever) to 4 (often). The total score of each part may range between 20 and 80, with higher scores indicating higher levels of anxiety. For our aim we used the Part 1 only to assess state anxiety, referring to the transitory emotional response involving unpleasant feelings of apprehension, tension, nervousness, and worry due to social isolation and the pandemic [Italian validated version (29)].

#### Relationship Questionnaire (RQ)

RQ (30) was used to measure attachment style. The RQ is a single-item measure made up of four short paragraphs, each describing a prototypical attachment pattern as it applies in close adult peer relationships. Participants are asked to rate their degree of correspondence to each prototype on a 7-point scale. The four attachment patterns (i.e., secure, preoccupied, fearful, and dismissing) are defined in terms of two dimensions: anxiety (i.e., a strong need for care and attention from attachment figures coupled with a pervasive uncertainty about the willingness of attachment figures to respond to such needs) and avoidance (i.e., discomfort with psychological intimacy and the desire to maintain psychological independence). The RQ paragraph describing fearful attachment reads as follows: “I am uncomfortable getting close to others. I want emotionally close relationships, but I find it difficult to trust others completely, or to depend on them. I worry that I will be hurt if I allow myself to become too close to others.” A cross-cultural study of the RQ conducted on a convenience sample of college students reported that the mean ± s.d. score for the Italian population was 3.09 ± 2.01 (31). For our purpose we decided to use the RQ categorically, by dividing the four attachment styles in “secure attachment” and, on the other hand “insecure attachment,” which includes fearful, preoccupied and dismissing attachment styles.

#### Parental Bonding Instrument (PBI)

PBI (13) was used to measure parental care experienced in childhood. The questionnaire is retrospective, meaning that adults (over 16 years) complete the measure for how they remember their parents during their first 16 years. The PBI includes two subscales assessing maternal and paternal care. The participants of this study were assigned to low care or high care groups based on their maternal and paternal care scores, using the suggested cut-off scores by Parker and Lipscombe (32). Individuals who reported scores lower than 27 on PBI maternal care scale and 24 on PBI paternal care scale were classified as low care individuals, whereas the others were considered high care individuals. The requirement of both maternal and parental care lower than cut-off in the low care group, was chosen in to include only individuals with severe lack of care, while those who received adequate maternal and paternal care were placed in the high care group. Whether one of the parents' care was not adequate, then individuals were included in an intermediate group.

The same group creation criterion was employed for the control dimension, whereas individuals who reported scores lower than 13.5 on PBI maternal care scale and 12.5 on PBI paternal care scale were classified as low control individuals, whereas the others were considered high control individuals. The requirement of both maternal and parental control higher than cut-off in the high control group, was chosen in to include only individuals who underwent overcontrol during childhood, while those who received adequate maternal and paternal control were placed in the low control group. Whether one of the parents' control was overt, then individuals were included in an intermediate group of control [Italian validated version (33)].

### Statistics

In order to assess the effects of the pandemic on psychopathological and stress-related variables repeated-measures analysis of variance (RM-ANOVA) was performed. Attachment style, parental care and parental control were used as categorial variables, while psychopathological and stress related ones were continuous measures (SCL-90-R subscales score, PSS score, STAI-Y state score). Attachment styles, parental care, and parental control were used as between-subject factors, whereas time as within-subject factors. Significant RM-ANOVAs were followed by *post-hoc* comparisons using either Duncan or Tukey HSD's test. Bonferroni's correction for multiple comparisons was also applied and the significance of the Bonferroni corrected *P*-value was also provided. Statistical analyses were carried out with the help of Statistica software Version 12.0 (StatSoft, Tulsa, OK, USA).

## Results

### Impact of the COVID-19 Pandemic Due to Confinement, Attachment Style, and Parental Bonding on Psychopathological Symptoms Evaluated by SCL-90-R

To determine whether the psychological parameters that were measured by SCL-90-R varied between phases 1 and 2, as a result of the restriction of social contact and mobility due to the pandemic, and whether these variations were modulated by attachment style and parental bonding, repeated measure ANOVA was performed for each SCL-90-R subscale.

**Figure 1:**
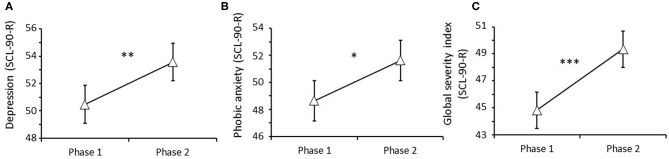
Effect of the pandemic on psychopathological symptoms measured by SCL-90-R. A significant deterioration in psychological conditions was observed during the confinement. A worsening in depressive symptoms **(A)**, phobic anxiety **(B)**, and general distress related to symptoms **(C)** was observed in the entire sample between phase 1 (before confinement) and 2 (during confinement). *P < 0.05, **P < 0.01, and ***P < 0.001.

First, the impact of attachment style, time, and their interaction was analyzed. Attachment style had a significant main effect for the following subscales: somatization [*F*_(1, 66)_ = 9.804, *P* = 0.002; Bonferroni corrected *P*-value = significant, s], obsessive-compulsivity [*F*_(1, 66)_ = 22.442, *P* < 0.001; Bonferroni corrected *P*-value = s], interpersonal sensitivity [*F*_(1, 66)_ = 19.132, *P* < 0.001; Bonferroni corrected *P*-value = s], depression [*F*_(1, 66)_ = 21.111, *P* < 0.001; Bonferroni corrected *P*-value = s], anxiety [*F*_(1, 66)_ = 10.530, *P* = 0.002; Bonferroni corrected *P*-value = s], hostility [*F*_(1, 66)_ = 4.59, *P* = 0.03; Bonferroni corrected *P*-value = not significant, ns], phobic anxiety [*F*_(1, 66)_ = 6.04, *P* = 0.02; Bonferroni corrected *P*-value = ns], paranoid ideation [*F*_(1, 66)_ = 15.105, *P* < 0.001; Bonferroni corrected *P*-value = s], psychoticism [*F*_(1, 66)_ = 18.147, *P* < 0.001; Bonferroni corrected *P*-value = s], and global severity index [*F*_(1, 66)_ = 13.770, *P* < 0.001; Bonferroni corrected *P*-value = s]. This effect comprised significantly higher scores for all these parameters in individuals with an insecure attachment style compared with those with secure attachment styles. Specifically, *post-hoc* comparisons performed on values obtained at phase 1 showed significantly higher scores for obsessive-compulsivity, interpersonal sensitivity, depression, anxiety, paranoid ideation, psychoticism, and global severity index in individuals with an insecure attachment style compared with those with secure attachment styles at this stage (Supplementary Table 1). Moreover, time had a significant main effect for depression [*F*_(1, 66)_ = 8.959, *P* = 0.004; Bonferroni corrected *P*-value = s], phobic anxiety [*F*_(1, 66)_ = 4.698, *P* = 0.03; Bonferroni corrected *P*-value = ns], and the general severity index [*F*_(1, 66)_ = 14.258, *P* < 0.001; Bonferroni corrected *P*-value = s], as evidenced by higher scores for these parameters in phase 2 vs. 1 for the entire sample. No attachment style x time interaction was detected (Figure 1; Supplementary Table 2).

The impact of parental care, time, and their interaction was also analyzed. A significant main effect of parental care was observed for the following subscales: somatization [*F*_(2, 65)_ = 3.435, *P* = 0.04; Bonferroni corrected *P*-value = ns], obsessive-compulsivity [*F*_(2, 65)_ = 5.177, *P* = 0.008; Bonferroni corrected *P*-value = ns], interpersonal sensitivity [*F*_(2, 65)_ = 7.944, *P* < 0.001; Bonferroni corrected *P*-value = s], depression [*F*_(2, 65)_ = 8.325, *P* = 0.003; Bonferroni corrected *P*-value = s], anxiety [*F*_(2, 65)_ = 4.204, *P* = 0.02; Bonferroni corrected *P*-value = ns], phobic anxiety [*F*_(2, 65)_ = 5.764, *P* = 0.004; Bonferroni corrected *P*-value = s], paranoid ideation [*F*_(2, 65)_ = 5.696, *P* = 0.005; Bonferroni corrected *P*-value = ns], psychoticism [*F*_(2, 65)_ = 8.455, *P* < 0.001; Bonferroni corrected *P*-value = s], and global severity index [*F*_(2, 65)_ = 4.888, *P* = 0.01; Bonferroni corrected *P*-value = ns]. This effect was reflected by significantly higher scores for all these parameters in individuals who received low compared with high and intermediate care. Specifically, *post-hoc* comparisons performed on values obtained at phase 1, showed significantly higher scores for obsessive-compulsivity, interpersonal sensitivity, depression, paranoid ideation, and psychoticism in individuals who received low compared with high care at this stage (Supplementary Table 3). Moreover, these comparisons showed significantly higher scores for interpersonal sensitivity, depression, and psychoticism, in individuals who received low compared with intermediate care at phase 1 (Supplementary Table 3). No parental care x time interaction was seen.

Finally, the impact of parental control, time, and their interaction was examined. Parental control had a significant main effect for the following subscales: somatization [*F*_(2, 65)_ =3.22, *P* = 0.04; Bonferroni corrected *P*-value = ns], interpersonal sensitivity [*F*_(2, 65)_ =9.263, *P* < 0.001; Bonferroni corrected *P*-value = s], depression [*F*_(2, 65)_ = 6.714, *P* = 0.002; Bonferroni corrected *P*-value = s], phobic anxiety [*F*_(2, 65)_ = 6.725, *P* = 0.002; Bonferroni corrected *P*-value = s], paranoid ideation [*F*_(2, 65)_ = 6.367, *P* = 0.002; Bonferroni corrected *P*-value = s], and psychoticism [*F*_(2, 65)_ = 4.125, *P* = 0.02; Bonferroni corrected *P*-value = ns]. This effect consisted of significantly higher scores for these parameters in individuals who experienced high vs. low and high-low parental control. Specifically, *post-hoc* comparisons performed on values obtained at phase 1, showed significantly higher scores for interpersonal sensitivity, depression, phobic anxiety, paranoid ideation, and psychoticism in individuals who received low compared with high control at this stage (Supplementary Table 4). Moreover, these comparisons showed significantly higher scores for interpersonal sensitivity in individuals who received high compared with intermediate control at phase 1 (Supplementary Table 4). No parental control x time interaction was observed.

### Impact of COVID-19 Pandemic Due to Confinement, Attachment Style, and Parental Bonding on Perceived Stress Evaluated by PSS

Repeated measure ANOVA was performed to determine whether perceived stress, as measured by the PSS, differed between phases 1 and 2 and whether these variations were modulated by attachment style, parental care and parental control.

**Figure 2:**
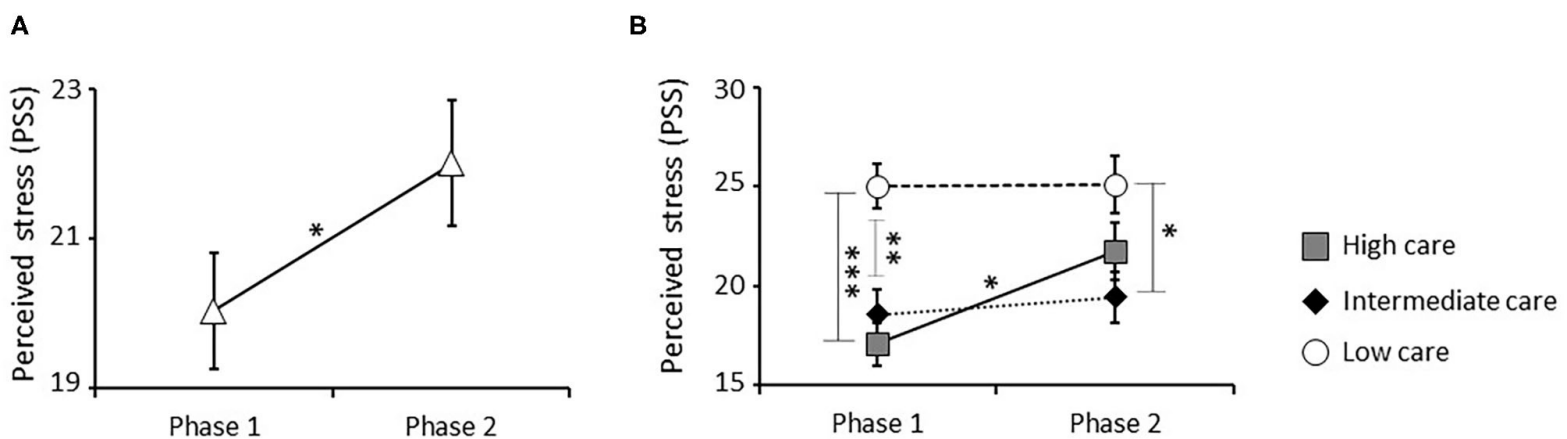
Effect of the pandemic and parental care on perceived stress measured by PSS. A significant increase in stress perception was detected in the entire sample between phase 1 (before confinement) and phase 2 (during confinement) **(A)**. Specifically, a significant interaction effect between parental care and time was observed. Low care individuals showed a significantly higher perceived stress than high and intermediate care groups at phase 1. Further, only high care individuals showed a significant increase in stress perception between phase 1 and 2, reaching levels similar to ones observed in the low care group **(B)** *P < 0.05, **P < 0.01, and ***P < 0.001.

First, the impact of attachment style, time, and their interaction was analyzed. We noted a significant main effect of attachment style [*F*_(1, 66)_ = 15.042, *P* < 0.001; Bonferroni corrected *P*-value = s], reflected by significantly higher PSS scores in individuals with an insecure vs. secure attachment style. Specifically, *post-hoc* comparisons performed on values obtained at phase 1, showed similar differences between groups at this stage (Supplementary Tables 1, 2). Moreover, time had a significant main effect [*F*_(1, 66)_ = 5.266, *P* = 0.025; Bonferroni corrected *P*-value = ns], consisting of higher PSS in phase 2 compared with phase 1 in the entire sample (Figure 2A). No significant attachment style x time interaction was detected.

Further, the impact of parental care, time, and their interaction was studied. There was a significant main effect of parental care [*F*_(2, 66)_ = 9.248, *P* < 0.001; Bonferroni corrected *P*-value = s], consisting of significantly higher PSS scores in individuals who received low vs. high or intermediate care. Specifically, *post-hoc* comparisons performed on values obtained at phase 1, showed similar differences among groups at this stage (Supplementary Table 3). Moreover, a significant interaction between parental care and time was also observed [*F*_(2, 65)_ = 3.243, *P* = 0.045; Bonferroni corrected *P*-value = ns], with only individuals who received high care showing a significant increase in PSS between phases 1 and 2 (Figure 2B).

Finally, the impact of parental control, time, and their interaction was analyzed. This analysis revealed a significant main effect of parental control [*F*_(2, 65)_ = 5.600, *P* = 0.006; Bonferroni corrected *P*-value = ns], comprising significantly higher PSS scores in individuals who received high vs. low control. Specifically, *post-hoc* comparisons performed on values obtained at phase 1, showed similar differences among groups at this stage (Supplementary Table 4). No significant effect of the parental control x time interaction was seen.

### Impact of COVID-19 Pandemic Due to Confinement, Attachment Style, and Parental Bonding on State Anxiety Evaluated by STAI-Y

To determine whether state anxiety, as measured by the STAI-Y, changed between phases 1 and 2 and whether these variations were modulated by attachment style, parental care, and parental control, repeated measure ANOVA was performed.

**Figure 3:**
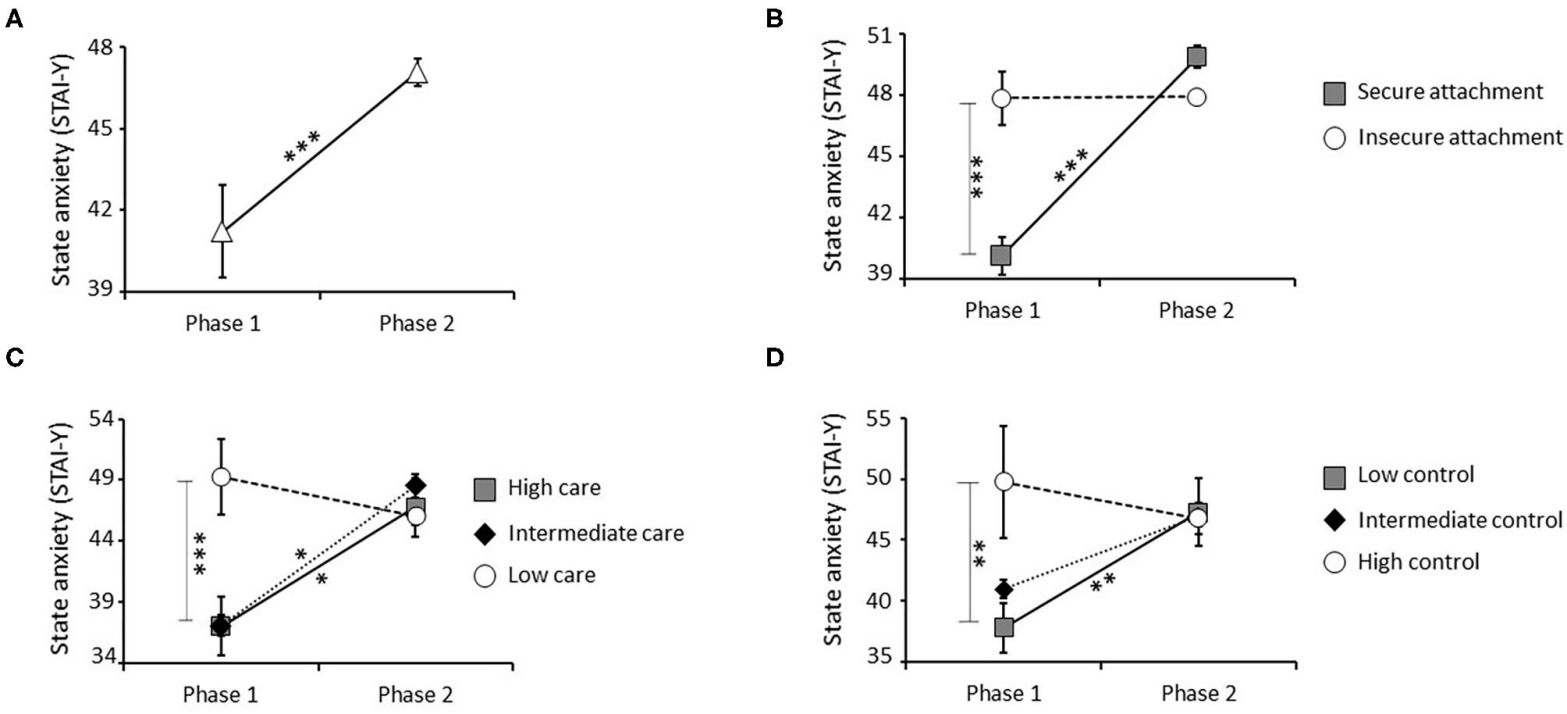
Effect of the pandemic, attachment style, parental care, and parental control on state anxiety measured by STAI-Y. A significant increase in state anxiety was detected in the entire sample between phase 1 (before confinement) and phase 2 (during confinement) **(A)**. A significant interaction effect between attachment style and time was also observed, with insecure individuals who showed higher state anxiety than secure subjects at phase 1. Further, only secure individuals showed a significant increase in state anxiety between phase 1 and 2, reaching levels similar to the ones observed in the insecure group **(B)**. A significant interaction effect between parental care and time was also observed, with low care individuals who showed higher state anxiety than high care and intermediate care groups at phase 1. Further, high care and intermediate care groups showed a significant increase in state anxiety between phase 1 and 2, reaching levels similar to the ones observed in the low care group **(C)**. Finally, a significant interaction effect between parental control and time was observed, with high control individuals who showed higher state anxiety than low control group at phase 1. Low control group showed a significant increase in state anxiety between phase 1 and 2, reaching levels similar to the ones observed in the high control group **(D)**. *P < 0.05, **P < 0.01, and ***P < 0.001.

First, the impact of attachment style, time, and their interaction was analyzed. This analysis revealed a significant main effect of attachment style [*F*_(1, 60)_ = 9.569, *P* = 0.003; Bonferroni corrected *P*-value = s], consisting of significantly higher state anxiety in individuals with insecure vs. secure attachment styles. Specifically, *post-hoc* analyses performed on values obtained at phase 1, showed similar difference between groups at this stage (Supplementary Tables 1, 2). Moreover, a significant main effect of time [*F*_(1, 60)_ = 22.256, *P* < 0.001; Bonferroni corrected *P*-value = s] was seen, based on higher levels of state anxiety in phase 2 compared with phase 1 in the entire sample (Figure 3A). A significant effect of the attachment style × time interaction was also detected [*F*_(1, 60)_ = 21.583, *P* < 0.001; Bonferroni corrected *P*-value = s], with only individuals with secure attachment showing a significant increase in state anxiety in phase 2 with respect to phase 1. As result of this effect, individuals with secure attachment did not differ from those with insecure attachment for this parameter in phase 2 (Figure 3B).

The influence of parental care, time, and their interaction was analyzed. Parental care had a significant main effect [*F*_(2, 59)_ = 6.163, *P* = 0.004; Bonferroni corrected *P*-value = s], consisting of significantly higher state anxiety in individuals who received low vs. high and intermediate care. Specifically, *post-hoc* comparisons performed on values obtained at phase 1, showed similar difference among groups at this stage (Supplementary Table 3). A significant interaction effect between parental care and time was also observed [*F*_(2, 59)_ = 6.822, *P* = 0.002; Bonferroni corrected *P*-value = s], with individuals who received high and intermediate care showing a significant increase in state anxiety between phases 1 and 2. No difference between phases 1 and 2 was noted in individuals who received low parental care (Figure 3C).

Finally, the impact of parental control, time, and their interaction was analyzed. Parental control had a significant main effect [*F*_(2, 59)_ = 4.331, *P* = 0.02; Bonferroni corrected *P*-value = ns], reflected by significantly higher state anxiety in individuals who experienced high compared with low control. Specifically, *post-hoc* comparisons performed on values obtained at phase 1, showed similar difference among groups at this stage (Supplementary Table 4). Finally, a significant parental control x time interaction effect was observed [*F*_(2, 59)_ = 3.341, *P* = 0.04; Bonferroni corrected *P*-value = ns] with individuals who received low or intermediate control showing significantly higher state anxiety between phases 1 and 2. No difference between phases 1 and 2 was seen in individuals who received high levels of parental control (Figure 3D).

## Discussion

This report is the first study to evaluate the impact of the COVID-19 pandemic on psychological symptoms in subjects who were psychometrically screened 6 months before restrictive measures were applied to contain it and at the end of this restriction period. This longitudinal experimental design allowed us to directly assess the effects of COVID-19 on mental health status and its correlates on the same subjects.

Moreover, a cross-cutting effect of the restrictive measures and the pandemic itself was noted. In particular, an increase in depressive symptoms, phobic anxiety, and general distress in relation to the symptoms themselves was detected in our sample during the lockdown. Moreover, greater perceived stress and state anxiety were observed. Consistent with these results, previous studies have shown that epidemics have detrimental effects on general mental health status (34, 35). During epidemics that have required quarantine measures, psychopathological manifestations have arisen, such as post-traumatic symptoms that belong to the symptomatological core in our study, as well as depressive, anxiety, and panic symptoms (34). Increased levels of depression, anxiety, and general distress were reported during the previous SARS outbreak (36) and other epidemics, such as swine flu and avian influenza (36). In line with these studies, evolutionary explanations show how the increased fear and emotional reactivity due to epidemics was selected by the natural selection in order to minimize infection risk (37).

Preliminary population studies have described similar results during the COVID-19 pandemic, with a wide presence of mood symptomatology (e.g., depression and anxiety) and phobia [for a review, see (38–40)]. Similarly, increased hostility, stress perception, and psychological distress have been reported (41).

In addition to the effects of the restrictive measures, we observed a significant effect of attachment style and parental care and control on psychopathological symptoms, perceived stress, and state anxiety parameters, with individuals with insecure attachment, low care, and high control exhibiting high scores on these scales. This effect was already visible at phase 1 and remained stable between phase 1 and phase 2 of the study in this group. However, the mean values obtained from this group were below the maximum scores that can be obtained on these scales (usually observable in clinical samples) excluding therefore the possibility of being in presence of a ceiling effect in these individuals. Overall, these results are consistent with the vast literature on the modulating effects of attachment style and parental care and control on the dimensions on several psychopathological traits, perceived stress, and state anxiety (42–47), again supporting the relevance of these events in psychopathological outcomes.

A notable aspect of this study was its detection of an interaction effect between attachment, parental bonding and confinement on perceived stress and state anxiety parameters. Specifically, we demonstrated that individuals with secure attachment, high parental care, and low control suffered more from the effects of confinement than their counterparts, who instead remained steadily high in terms of perceived stress and state anxiety between the first and second phases of the study. One interpretation of this result is that the lockdown/confinement was not stressful enough to increase stress perception and state anxiety in insecure, low care individuals, who are accustomed to dealing with large amounts of socio-relational stress.

Another explanation, connected in part to the previous hypothesis, attributes the absence of an effect of the confinement measures in the insecure, low care group to “malfunction” of their stress response system, which when well-functioning in secure, high care subjects elicits increased perceived stress and state anxiety under such stressful conditions. Support for this hypothesis comes from studies that have consistently reported psycho-pathological symptoms in individuals who have experienced low levels of parental care and/or dysfunctional parent-child attachment at an early age (44–46). This ability to cope with environmental changes is pivotal for an individual's survival and matures as a result of the events that are experienced at an early age (48, 49). These results implicate disparate sensitivities to environmental changes in the high- and low care groups during the lockdown, the former of which shows the greatest flexibility in the response to environmental stimulation, suggesting adequate ability to cope with stress in high care individuals, which is not observable in the low care group.

This study has several limitations: (1) few subjects were enrolled, and (2) the sample was mainly composed of female students, and this reduces the generalizability of our results to the general population. About the low number of subjects we want to underlie that as being a longitudinal study comparing the first phase, 6 months before COVID-19 pandemic, with the second phase, during the COVID-19-due confinement, we could not decide the number of subjects to test because of the unpredictability of the pandemic. The low number of subjects partially influenced the low *p*-value significance obtained in our study, and some results were not significant after Bonferroni correction for multiple testing. However, although we decided to provide *p*-value significance with and without Bonferroni correction in the manuscript, we kept discussing and presenting our results as significant considering the *p*-value without Bonferroni correction. This has been done considering that the results obtained from this longitudinal study are extremely relevant to understand the psychological consequences of being exposed to COVID-19-due confinement, although the obtained data do not meet a statistical precision criterion due to the aforementioned limitations.

Our results obtained raise 3 notable questions that warrant further investigation: (1) Does the psychological suffering in the entire sample (depressive, phobic anxiety, and hostility symptoms) return to normal levels after suspension of the confinement, or does this event induce a more structured disease? (2) Does perceived stress and state anxiety levels in secure, high care individuals return to normal levels after suspension of the confinement? (3) Is it possible that insecure, low care individuals, although they do not exhibit increased perceived stress and state anxiety during confinement, develop this symptomatology several weeks after the end of confinement, supporting the hypothesis of a delayed coping response than in the absence of such a response?

Future longitudinal studies should address these questions to determine the need for psychological interventions in mitigating the psychological impacts of this pandemic.

## Data Availability Statement

The raw data supporting the conclusions of this article will be made available by the authors, without undue reservation.

## Ethics Statement

The studies involving human participants were reviewed and approved by Ethical Committee of the Department of Dynamic and Clinical Psychology, Sapienza University of Rome. The patients/participants provided their written informed consent to participate in this study.

## Author Contributions

SB and CP collected the data. SB and VC analyzed the data and designed the figures. SB, RT, and VC wrote the manuscript. All authors contributed to the article and approved the submitted version.

## Conflict of Interest

The authors declare that the research was conducted in the absence of any commercial or financial relationships that could be construed as a potential conflict of interest.
