# Supplementary material for: Psychological Health Issues Subsequent to SARS-Cov 2 Restrictive Measures: The Role of Parental Bonding and Attachment Style
Source: Front Psychiatry. 2020 Nov 4;11:589444. doi: 10.3389/fpsyt.2020.589444 (PMC7672158; doi:10.3389/fpsyt.2020.589444)
Supplement: Supplementary file 1 [file Table_1.docx]

Supplementary Table 1

|  | **Phase 1** | |  | **Phase 2** | | | |  | |
| --- | --- | --- | --- | --- | --- | --- | --- | --- | --- |
|  | *Secure Attachment* | *Insecure Attachment* | *P value* | | *Secure Attachment* | *Insecure Attachment* | *P value* | |  |
|  | *(N = 24)* | *(N = 44)* |  | | *(N = 24)* | *(N = 44)* |  | |  |
| **SCL-90-**   - somatization | 45.66 ± 1.28 | 51.30 ± 1.51 | n.s. | | 45.16 ± 1.98 | 52.54 ± 1.60 | p<0.05 | |  |
| - obsessive-compulsivity | 42.92 ± 1.40 | 54.16 ± 1.75 | p<0.001 | | 44.13 ± 1.91 | 56.25 ± 1.76 | p<0.001 | |  |
| - interpersonal sensitivity | 40.88 ± 1.24 | 51.20 ± 1.58 | p<0.001 | | 42.50 ± 1.41 | 50.68 ± 1.60 | p<0.01 | |  |
| - depression | 43.21 ± 1.09 | 54.43 ± 1.83 | p<0.001 | | 46.63 ± 1.75 | 57.39 ± 1.68 | p<0.001 | |  |
| - anxiety | 41.17 ± 1.70 | 53.41 ± 1.68 | p <0.05 | | 46.46 ± 1.93 | 54.48 ± 1.77 | p<0.05 | |  |
| - hostility | 42.54 ± 0.78 | 48.23 ± 1.35 | p<0.05 | | 46.75 ± 1.74 | 48.45 ± 1.25 | n.s. | |  |
| - phobic anxiety | 44.92 ± 1.25 | 50.68 ± 1.53 | n.s. | | 48.25 ± 1.82 | 53.48 ± 1.96 | n.s. | |  |
| - paranoid ideation | 38.16 ± 1.31 | 46.07 ± 1.37 | p<0.01 | | 39.13 ± 1.41 | 45.34 ± 1.41 | p<0.05 | |  |
| - psychoticism | 42.42 ± 0.76 | 53.05 ± 1.56 | p<0.001 | | 44.36 ± 1.54 | 51.66 ± 1.57 | p<0.05 | |  |
| - GSI | 39.54 ± 1.21 | 47.73 ± 1.84 | p<0.05 | | 43.29 ± 1.72 | 52.64 ± 1.66 | p<0.01 | |  |
| **PSS** | 16.67 ± 1.04 | 21.86 ± 0.96 | p<0.01 | | 18.67 ± 1.55 | 23.82 ± 0.89 | p<0.01 | |  |
| **STAI-Y state** | 32.91 ± 1.55 | 45.76 ± 2.17 | p<0.001 | | 49.18 ± 0.88 | 45.9 ± 0.53 | n.s. | |  |
