# Supplementary material for: Psychological Health Issues Subsequent to SARS-Cov 2 Restrictive Measures: The Role of Parental Bonding and Attachment Style
Source: Front Psychiatry. 2020 Nov 4;11:589444. doi: 10.3389/fpsyt.2020.589444 (PMC7672158; doi:10.3389/fpsyt.2020.589444)
Supplement: Supplementary file 2 [file Table_2.DOCX]

Supplementary Table 2

| **SCL-90-R** | **Phase 1** | **Phase 2** |
| --- | --- | --- |
| - Depression | 50.47 ± 1.40* | 53.59 ± 1.39 |
| - Phobic Anxiety | 48.65 ± 1.12 | 51.63 ± 1.44 |
| - Global Severity Index | 44.84 ± 1.34 | 49.34 ± 1.35 |
| **PSS** | 20.03 ± 0.78 | 22.00 ± 0.84 |
| **STAI-Y state** | 41.21 ± 1.69 | 47.06 ± 0.5 |

*mean + st. err.
