# Supplementary material for: Psychological Health Issues Subsequent to SARS-Cov 2 Restrictive Measures: The Role of Parental Bonding and Attachment Style
Source: Front Psychiatry. 2020 Nov 4;11:589444. doi: 10.3389/fpsyt.2020.589444 (PMC7672158; doi:10.3389/fpsyt.2020.589444)
Supplement: Supplementary file 3 [file Table_3.DOCX]

Supplementary Table 3

|  | **Phase 1** | | |  | **Phase 2** | | |  |
| --- | --- | --- | --- | --- | --- | --- | --- | --- |
|  | **Parental Care** | | |  | **Parental Care** | | |  |
|  | *Low* | *Intermediate* | *High* | *P value* | *Low* | *Intermediate* | *High* | *P value* |
|  | *(N = 21)* | *(N = 23)* | *(N = 24)* |  | *(N = 21)* | *(N = 23)* | *(N = 24)* |  |
| **SCL-90-R**   - Somatization | 52.29 ± 2.34 | 48.17 ± 1.68 | 47.69 ± 1.75 | n.s. | 54.76 ± 2.57 | 49.04 ± 1.98 | 46.58 ± 2.01 | n.s. |
| - Obsessive-Compulsivity | 56.95 ± 2.26 | 47.56 ± 2.29 | 46.79 ± 2.20 | #p<0.05 | 57.38 ± 2.71 | 47.74 ± 2.47 | 51.29 ± 2.32 | n.s. |
| - Interpersonal Sensitivity | 55.47 ± 2.44 | 44.48 ± 1.91 | 43.58 ± 1.34 | #p<0.001  *p<0.05 | 52.24 ± 2.41 | 44.91 ± 1.67 | 46.67 ± 2.11 | n.s. |
| - Depression | 58.43 ± 2.57 | 47.91 ± 2.22 | 45.96 ± 1.78 | #p<0.01  *p<0.05 | 60.05 ± 2.37 | 49.78 ± 2.18 | 51.58 ± 2.25 | *p<0.05 |
| - Anxiety | 56.19 ± 2.34 | 49.91 ± 2.42 | 47.08 ± 1.66 | n.s. | 55.52 ± 2.63 | 51.57 ± 2.49 | 48.33 ± 2.06 | n.s. |
| - Hostility | 48.81 ± 1.79 | 46.48 ± 2.02 | 43.71 ± 1.04 | n.s. | 49.00 ± 2.02 | 47.09 ± 1.42 | 47.58 ± 1.85 | n.s. |
| - Phobic Anxiety | 53.90 ± 2.44 | 46.70 ± 1.46 | 45.92 ± 1.57 | n.s. | 56.90 ± 3.24 | 48.78 ± 2.13 | 49.75 ± 1.88 | n.s. |
| - Paranoid Ideation | 48.81 ± 1.88 | 41.48 ± 1.96 | 40.17 ± 1.39 | #p<0.05 | 46.57 ± 2.19 | 41.91 ± 1.91 | 41.33 ± 1.48 | n.s. |
| - Psychoticism | 55.81 ± 2.36 | 47.91 ± 2.11 | 44.92 ± 1.11 | #p<0.001  *p<0.01 | 54.90 ± 2.37 | 46.78 ± 1.87 | 46.21 ± 1.73 | #p<0.05  *p<0.05 |
| - GSI | 50.05 ± 2.95 | 43.48 ± 2.30 | 41.58 ± 1.38 | n.s. | 55.00 ± 2.57 | 46.57 ± 2.08 | 47.04 ± 2.07 | n.s. |
| **PSS** | 25 ± 1.13 | 18.57 ± 1.27 | 17.08 ± 1.07 | #p<0.001  *p<0.01 | 25.1 ± 1.45 | 19.43 ± 1.28 | 21.75 ± 1.45 | *p<0.05 |
| **STAI-Y state** | 49.29 ± 3.08 | 37.11 ± 2.40 | 37.04 ± 2.42 | #p<0.001  *p<0.001 | 46.1 ± 0.80 | 48.56 ± 0.95 | 46.78 ± 0.81 | n.s. |

# Low vs High

§ Intermediate vs High

* Intermediate vs Low

n.s. not significant
