# Supplementary material for: Psychological Health Issues Subsequent to SARS-Cov 2 Restrictive Measures: The Role of Parental Bonding and Attachment Style
Source: Front Psychiatry. 2020 Nov 4;11:589444. doi: 10.3389/fpsyt.2020.589444 (PMC7672158; doi:10.3389/fpsyt.2020.589444)
Supplement: Supplementary file 4 [file Table_4.DOCX]

Supplementary Table 4

|  | | **Phase 1** | | | | |  | | **Phase 2** | | | | | |  | |  |
| --- | --- | --- | --- | --- | --- | --- | --- | --- | --- | --- | --- | --- | --- | --- | --- | --- | --- |
|  | | **Parental Control** | | | | |  | | **Parental Control** | | | | | |  | |  |
|  | | *Low* | *Intermediate* | | *High* | *P value* | | | | *Low* | *Intermediate* | | | *High* | *P value* | | |
|  | *(N = 34)* | | *(N = 21)* | *(N = 13)* | | | |  | | *(N = 34)* | | *(N = 21)* | *(N = 13)* | | |  |  |
| **SCL-90-**   - Somatization | 48.09 ± 1.40 | | 48.33 ± 2.01 | 54.08 ± 3.02 | | | | n.s. | | 48.44 ± 1.61 | | 48.71 ± 2.21 | 55.85 ± 3.80 | | | n.s. |  |
| - Obsessive-compulsivity | 47.09 ± 1.64 | | 50.52 ± 2.75 | 57.77 ± 3.19 | | | | n.s. | | 49.71 ± 1.89 | | 52.81 ± 2.56 | 56.54 ± 4.34 | | | n.s. |  |
| - Interpersonal sensitivity | 43.26 ± 1.21 | | 48.10 ± 1.92 | 57.92 ± 3.60 | | | | #p<0.001  §p<0.05 | | 44.94 ± 1.43 | | 48.71 ± 2.11 | 53.77 ± 3.58 | | | n.s. |  |
| - Depression | 46.41 ± 1.61 | | 51.05 ± 2.40 | 60.15 ± 3.45 | | | | #p<0.01 | | 50.79 ± 1.85 | | 53.67 ± 2.16 | 60.77 ± 3.66 | | | n.s. |  |
| - Anxiety | 48.59 ± 1.65 | | 50.48 ± 2.44 | 57.38 ± 3.09 | | | | n.s. | | 50.32 ± 1.90 | | 51.71 ± 2.46 | 55.00 ± 3.71 | | | n.s. |  |
| - Hostility | 45.29 ± 1.29 | | 47.29 ± 2.15 | 46.92 ± 1.62 | | | | n.s. | | 47.76 ± 1.63 | | 47.43 ± 1.50 | 48.77 ± 2.18 | | | n.s. |  |
| - Phobic anxiety | 46.41 ± 1.15 | | 47.67 ± 1.93 | 56.08 ± 3.41 | | | | #p<0.05 | | 49.79 ± 1.89 | | 49.67 ± 2.05 | 59.62 ± 4.15 | | | #p<0.05 |  |
| - Paranoid ideation | 40.71 ± 1.24 | | 43.19 ± 2.06 | 50.15 ± 2.66 | | | | #p<0.05 | | 40.41 ± 1.14 | | 44.71 ± 2.02 | 47.77 ± 3.27 | | | n.s. |  |
| - Psychoticism | 46.68 ± 1.45 | | 49.43 ± 2.29 | 55.92 ± 2.89 | | | | #p<0.05 | | 47.26 ± 1.64 | | 48.67 ± 2.01 | 54.62 ± 3.20 | | | n.s. |  |
| - Global severity index | 43.62 ± 1.55 | | 44.29 ± 2.53 | 48.92 ± 4.07 | | | | n.s. | | 47.12 ± 1.68 | | 49.10 ± 2.23 | 55.54 ± 3.92 | | | #p<0.05 |  |
| **PSS** | 17.62 + 1.07 | | 20.67 + 0.94 | 25.31 + 1.86 | | | | #p<0.01 | | 20.59 + 1.38 | | 22.76 + 0.85 | 24.46 + 2.04 | | | n.s. |  |
| **STAI-Y state** | 37.84 ± 2.02 | | 41.00 ± 2.78 | 49.77 ± 4.6 | | | | #p<0.01 | | 47.25 ± 0.70 | | 46.94 ± 0.88 | 46.77 ± 1.25 | | | n.s. |  |

# Low vs High

§ Intermediate vs High

* Intermediate vs Low

n.s. not significant
