# Supplementary material for: Psychological Health Issues Subsequent to SARS-Cov 2 Restrictive Measures: The Role of Parental Bonding and Attachment Style
Source: Front Psychiatry. 2020 Nov 4;11:589444. doi: 10.3389/fpsyt.2020.589444 (PMC7672158; doi:10.3389/fpsyt.2020.589444)
Supplement: Supplementary file 5 [file Table_5.DOCX]

**SUPPLEMENTARY MATERIALS**

# SUPPLEMENTARY TABLE LEGENDS

**Supplementary Table 1**

Mean scores, standard errors, and p values (post-hoc comparisons) for SCL-90-R subscales, Perceived Stress Scale, and State-Trait Anxiety Inventory – Y (state anxiety), in the different groups classified on the bases of the attachment style (secure and insecure) during Phase 1 and Phase 2.

**Supplementary Table 2**

Mean score and standard error for SCL-90-R subscales, Perceived Stress Scale, and State-Trait Anxiety Inventory – Y (state anxiety) of the all sample during Phase 1 and Phase 2.

**Supplementary Table 3**

Mean score, standard error and p value for within comparisons for SCL-90-R subscales, Perceived Stress Scale and State-Trait Anxiety Inventory – Y (state anxiety), in the different groups classified on the bases of the perceived parental care (low, intermediate and high care) during Phase 1 and Phase 2.

**Supplementary Table 4**

Mean score, standard error and p value for within comparisons for SCL-90-R subscales, Perceived Stress Scale and State-Trait Anxiety Inventory – Y (state anxiety), in the different groups classified on the bases of the perceived parental control (low, intermediate and high control) during Phase 1 and Phase 2.
